# Supplementary material for: Individuality and stability of the koala (Phascolarctos cinereus) faecal microbiota through time
Source: PeerJ. 2023 Jan 23;11:e14598. doi: 10.7717/peerj.14598 (PMC9879153; doi:10.7717/peerj.14598)
Supplement: Supplemental Information 1 [file peerj-11-14598-s001.docx]

| **Eucalypt species found at Mountain Lagoon** | | | |
| --- | --- | --- | --- |
| **Species name** | **Subgenus** | **Common name** | **PSMs** |
| *Corymbia gummiferea* |  | Red Bloodwood | Phenolics, terpenes |
| *Eucalyptus piperita* | *Eucalyptus* | Sydney peppermint | Phenolics, terpenes, UBFs |
| *Eucalyptus agglomerata* | *Eucalyptus* | Blue leaved Stringybark | Phenolics, terpenes, UBFs |
| *Eucalyptus globoidea* | *Eucalyptus* | White Stringybark | Phenolics, terpenes, UBFs |
| *Angophora costata* |  | Smooth-barked apple | Phenolics, terpenes |
| *Syncarpia glomulifera* |  | Turpentine | Terpenes |
| *Eucalyptus cypellocarpa* | *Symphyomyrtus* | Mountain Grey Gum | Phenolics, FPCs, terpenes |
| *Eucalyptus beyeriana* | *Symphyomyrtus* | Narrow-leafed Ironbark | Phenolics, FPCs, terpenes, |
| *Eucalyptus paniculata* | *Symphyomyrtus* | Grey Ironbark | Phenolics, FPCs, terpenes |
| *Eucalyptus saligna* | *Symphyomyrtus* | Sydney blue gum | Phenolics, FPCs, terpenes |
| *Eucalyptus deanei* | *Symphyomyrtus* | Mountain blue gum | Phenolics, FPCs, terpenes |
| *Eucalyptus punctata* | *Symphyomyrtus* | Grey gum | Phenolics, FPCs, terpenes |
