# Supplementary figures and images for: Individuality and stability of the koala (Phascolarctos cinereus) faecal microbiota through time
Source: PeerJ. 2023 Jan 23;11:e14598. doi: 10.7717/peerj.14598 (PMC9879153; doi:10.7717/peerj.14598)

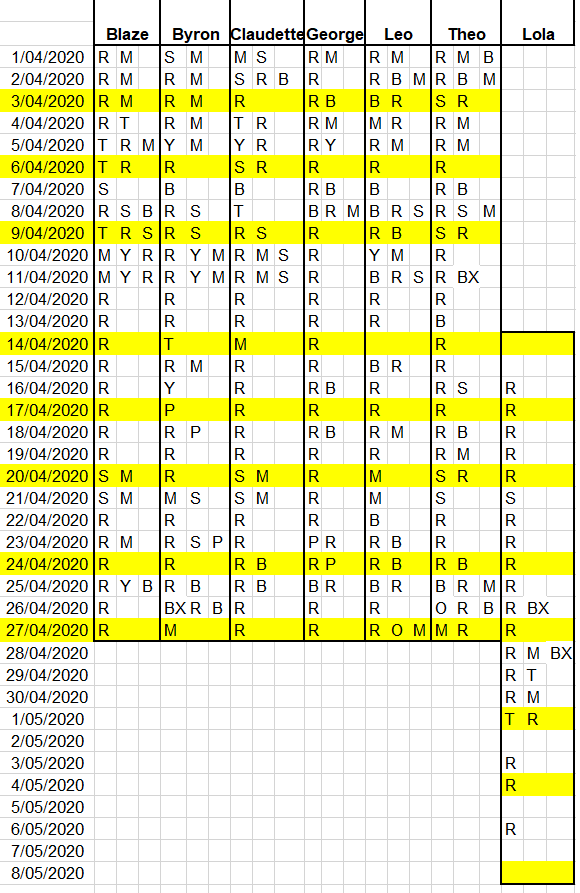

Supplement: Supplemental Information 2 — Yellow shading represents the days that faecal samples were collected. M = Manna (Eucalyptus viminalis) R = Red (Eucalyptus camaldulensis) T = Tassie Blue (Eucalyptus globulus) Y = Yate (Eucalyptus occidentalis) P = Platypus (Eucalyptus platypus) S = Stringybark (Eucalyptus obliqua) B = SA Blue (Eucalyptus leucoxylon) BX = Baxteri (Eucalyptus baxteri) O = Messmate (Eucalyptus obliqua). [file peerj-11-14598-s002.docx]

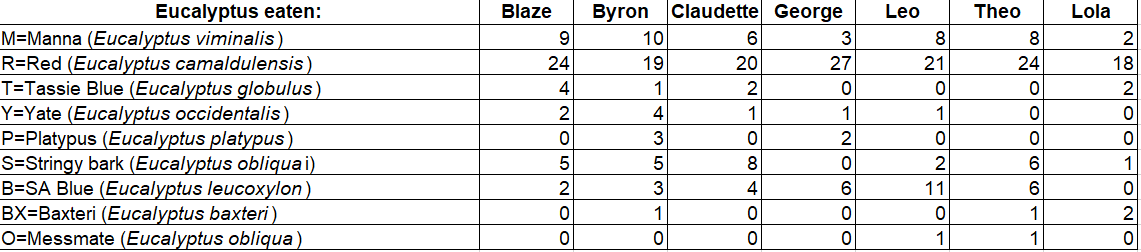

Supplement: Supplemental Information 3 — Sum of eucalyptus species eaten by Cleland koalas through the course of the study. [file peerj-11-14598-s003.docx]

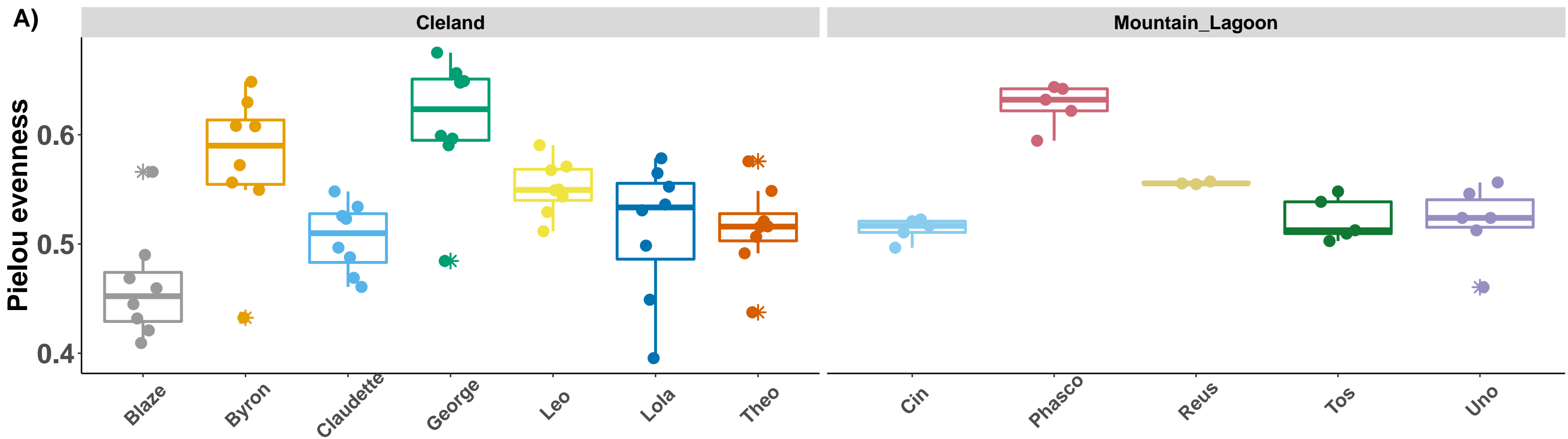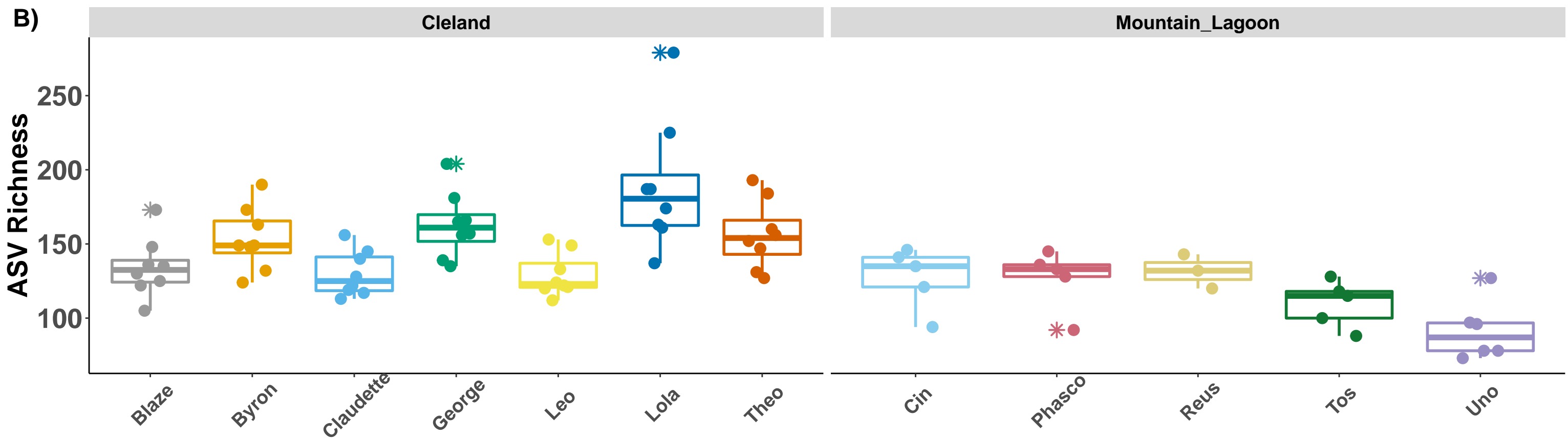

Supplement: Supplemental Information 5 — Pielou’s evenness (A) and ASV richness (B). [file peerj-11-14598-s005.pdf]

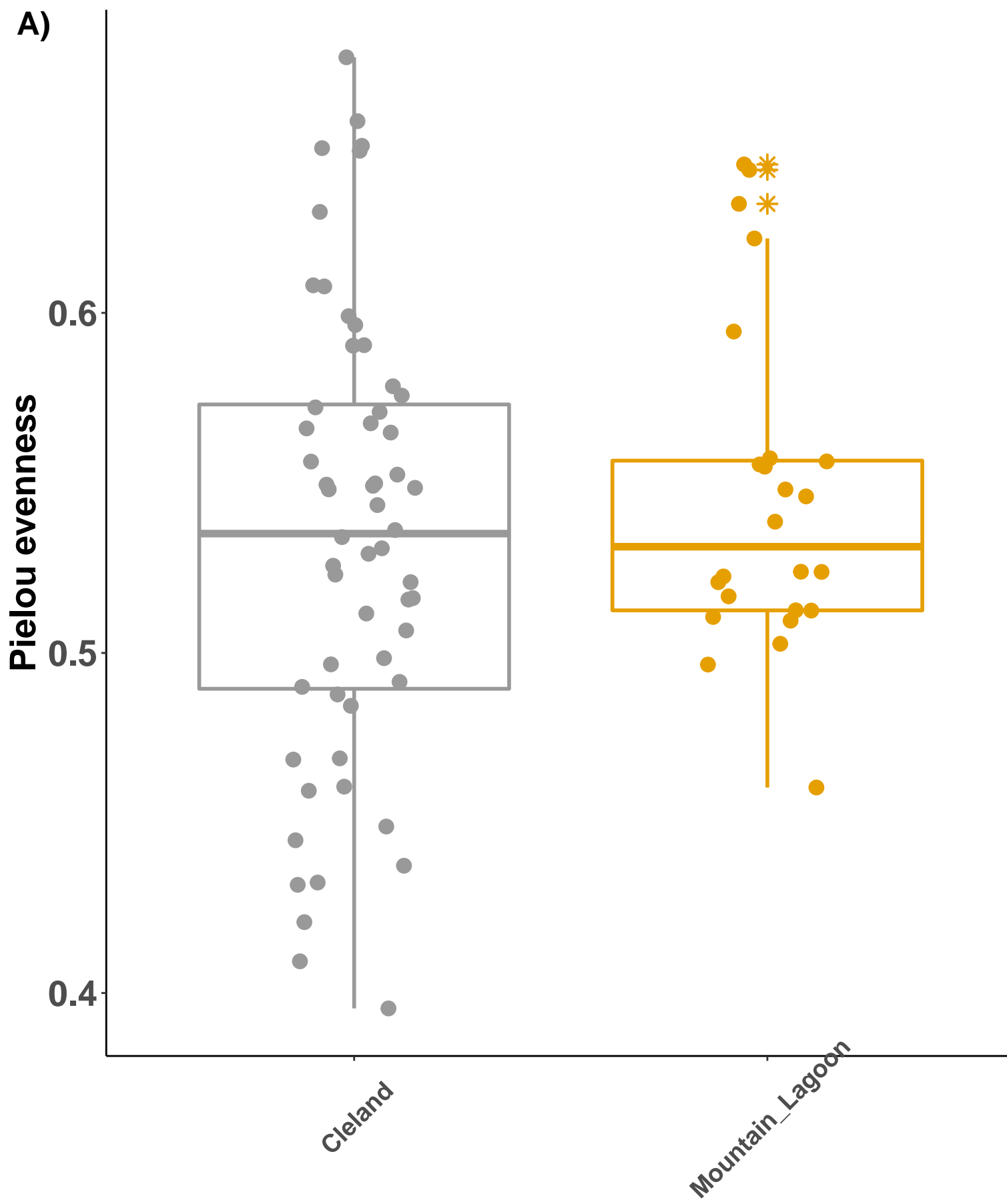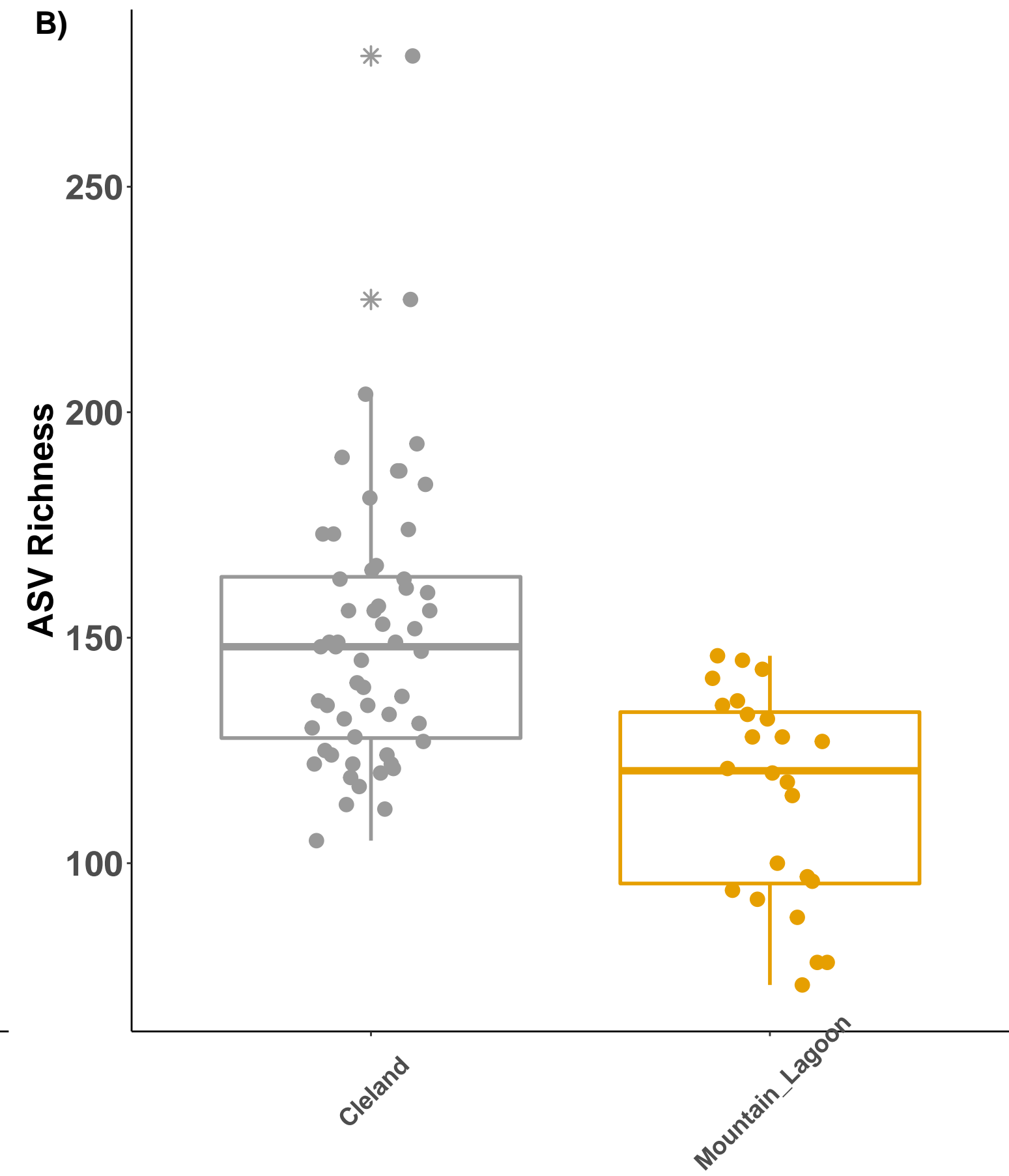

Supplement: Supplemental Information 6 — Pielou’s evenness (A) and ASV richness (B). [file peerj-11-14598-s006.pdf]

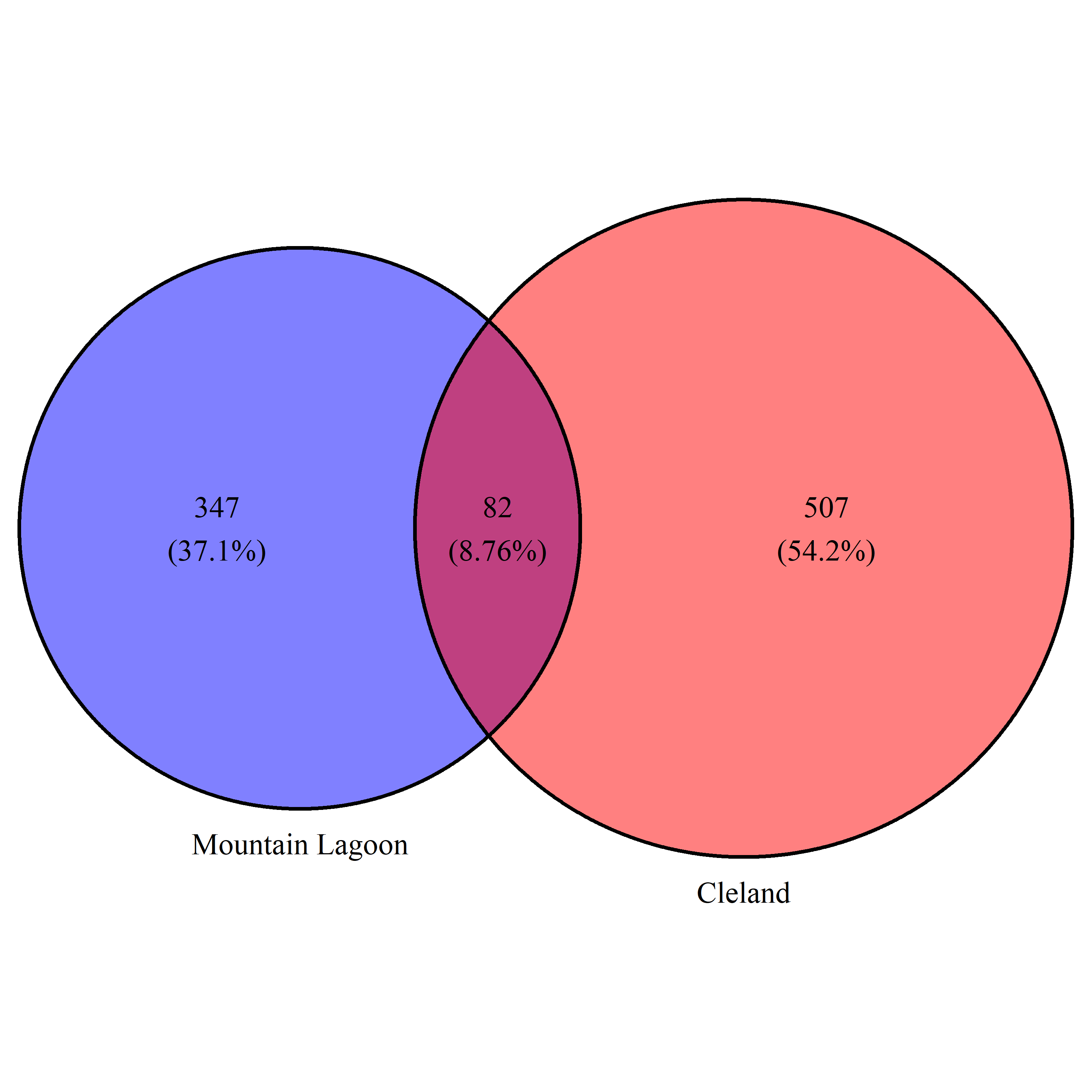

Supplement: Supplemental Information 7 — Percentages represent the proportion of total ASVs for each region. [file peerj-11-14598-s007.png]

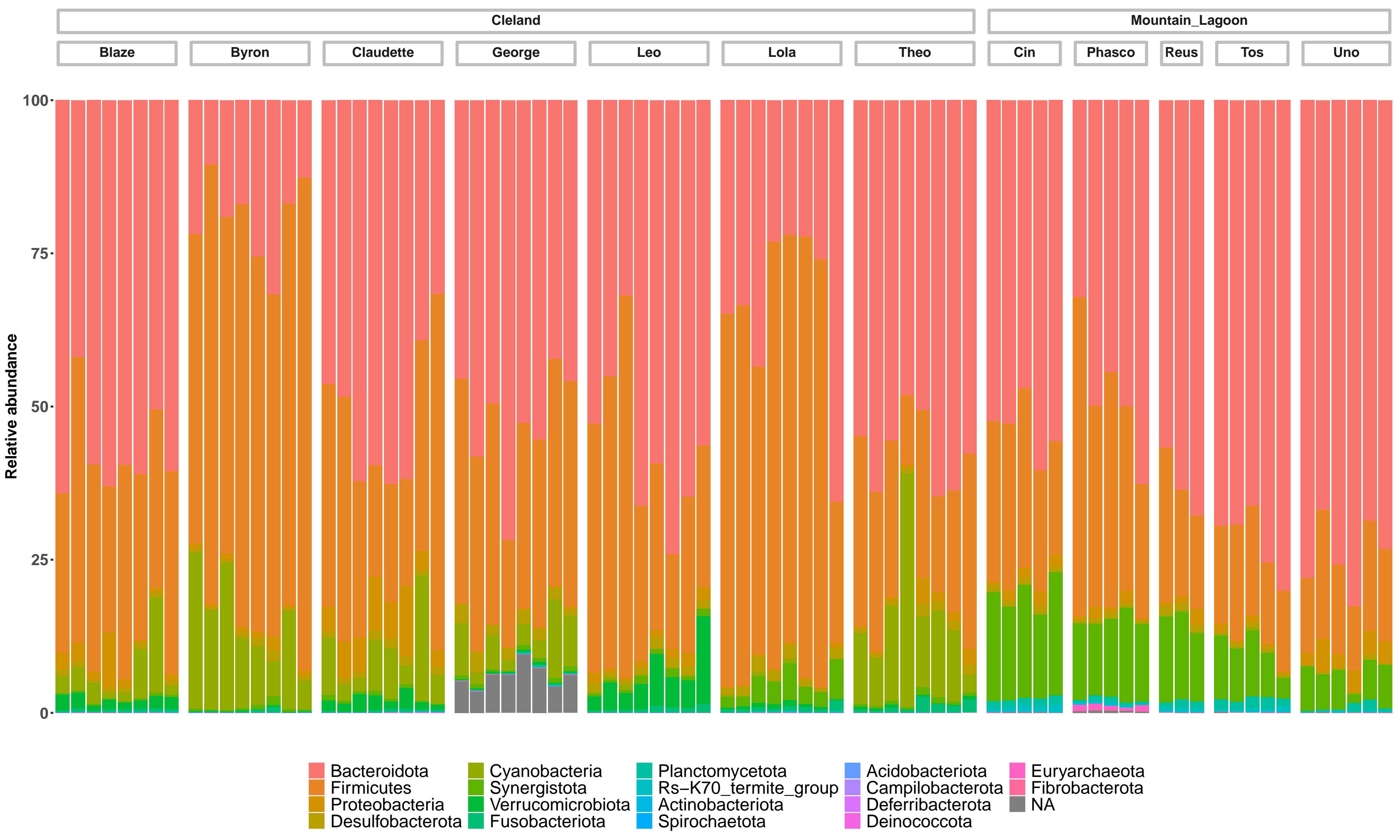

Supplement: Supplemental Information 8 — Relative abundance of different bacterial and archaeal phyla. [file peerj-11-14598-s008.pdf]
