# Supplementary material for: Individuality and stability of the koala (Phascolarctos cinereus) faecal microbiota through time
Source: PeerJ. 2023 Jan 23;11:e14598. doi: 10.7717/peerj.14598 (PMC9879153; doi:10.7717/peerj.14598)
Supplement: Supplemental Information 4 [file peerj-11-14598-s004.docx]

| **Highly prevalent and abundant ASVs** | | | |
| --- | --- | --- | --- |
| **ASV hash** | **Taxonomy** | **Mean relative abundance** | **Koala population** |
| 593a5c45dd38f6d9ec369b9b2679997f | *Tannerellaceae: Parabacteroides* | 18.6% (± 15.3%) | Cleland |
| ce3b37d15aee37e4213c2d13ad8b1b1a | *Paludibacteraceae* | 15.6% (± 9.9%) | Mountain Lagoon |
| f3521f355b29e7f7c878d616495def33 | *Tannerellaceae; Parabacteroides* | 20.7% (± 11.2%) | Mountain Lagoon |
